# Clinical prediction model of pathological response following neoadjuvant chemoradiotherapy for rectal cancer

**DOI:** 10.1038/s41598-022-10974-7

**Published:** 2022-05-03

**Authors:** Jung Kyong Shin, Jung Wook Huh, Woo Yong Lee, Seong Hyeon Yun, Hee Cheol Kim, Yong Beom Cho, Yoon Ah Park

**Affiliations:** Department of Surgery, Samsung Medical Center, Sungkyunkwan University School of Medicine, 81 Irwon-ro, Gangnam-gu, Seoul, 06351 Korea

**Keywords:** Colorectal cancer, Surgical oncology

## Abstract

Patients with pathologic complete response (pCR) achievement can consider local excision or “watch and wait” strategy instead of a radical surgery. This study analyzed the predictive factors of pCR in rectal cancer patients who underwent radical operation after neoadjuvant chemoradiotherapy (nCRT). This study also analyzed the recurrence patterns in patients who achieved pCR and the oncologic outcomes and prognostic factors by ypStage. Between 2000 and 2013, 1,089 consecutive rectal cancer patients who underwent radical resection after nCRT were analyzed. These patients were classified into two groups according to pCR. The clinicopathologic and oncologic outcomes were analyzed and compared between the two groups. Multivariate analysis was conducted on factors related to pCR. The proportion of patients achieving pCR was 18.2% (n = 198). The pCR group demonstrated earlier clinical T and N stages, smaller tumor size, better differentiation, and a lower percentage of circumferential resection margin (CRM) involvement than did the non-pCR group. The prognostic factors associated with poorer disease-free survival were high preoperative carcinoembryonic antigen levels, non-pCR, poor histology, lymphatic/perineural invasion, and involvement of CRM. Multivariate analysis revealed that clinical node negativity, tumor size < 4 cm, and well differentiation were significant independent clinical predictors for achieving pCR. Patients with pCR displayed better long-term outcomes than those with non-pCR. The pCR-prediction model, based on predictive factors, is potentially useful for prognosis and for prescribing a treatment strategy in patients with advanced rectal cancer who need nCRT.

## Introduction

According to the National Comprehensive Cancer Network guidelines, patients with advanced rectal cancer are initially treated with neoadjuvant chemoradiotherapy (nCRT)^1^. The purpose of nCRT in rectal cancer patients is to increase the rate of radical resection and sphincter-saving and decrease the rate of local recurrence^2^. The incidence of a pathologic complete response (pCR) ranges from 10 to 30% and has been associated with favorable oncological outcomes^3–7^. Total mesorectal excision (TME) is considered the standard method of rectal cancer surgery. It may lead to urinary and sexual dysfunction and the possibility of stoma formation^8,9^. Recently, studies have reported on patients who achieved clinical complete response (cCR) after nCRT using the “watch-and-wait” approach instead of destructive surgery^3,10,11^. Therefore, it is imperative to determine the predictive factors for pCR in order to select patients eligible for the “watch-and-wait” approach. It is also necessary to determine whether the oncologic outcomes of patients with pCR are actually better than those of those with non-pCR. Several studies have reported on predictive factors for pCR and the oncological outcomes of patients who achieved pCR^12^. However, few studies predict pCR based on clinical factors.

The purpose of this study was to analyze the predictive clinical factors of pCR in patients with rectal cancer who had undergone radical surgery after nCRT. This study also analyzed the recurrence patterns in patients who had achieved pCR as well as the oncologic outcomes and prognostic factors by ypStage.

## Results

### Clinicopathologic characteristics of the patients according to the pCR

Among the 1,089 patients in this study, the proportion of pCR patients was 18.2% (n = 198). The clinicopathological features of all patients are shown in Table 1. There were no significant differences in age, sex, preoperative carcinoembryonic antigen (CEA) level, TME grade, and vascular invasion between the pCR and non-pCR groups. However, there were statistically significant differences in histology (*P* < 0.001), clinical T stage (*P* = 0.007), and N stage (*P* = 0.030). There was no difference between the two groups in preoperative treatment-related factors. Similar results were shown between the two groups at the interval between nCRT and surgery (*P* = 0.710), neoadjuvant chemotherapy regimen (*P* = 0.683), and neoadjvuant radiotherapy dose (*P* = 0.774).

**Table 1:** Clinicopathologic characteristics of the patients.

	ypCR (n = 198)	Non-ypCR (n = 891 )	*P*
Age ( years, median±SD)	55±11	56±11	0.264
BMI (kg/m^2^)	24.7±3.0	23.8±3.0	<0.001
Gender, n(%)			0.748
Male	133 (67.2)	609 (68.4)	
Female	65 (32.8)	282 (31.6)	
CEA (ng/mL)			0.151
<5	192 (97.0)	842 (94.5)	
≥5	6 (3.0)	49 (5.5)	
Mean height from AV (cm)	4.4±1.9	4.3±2.3	0.187
Clinical T stage, n(%)			0.007
cT1	2 (1.0)	3(0.3)	
cT2	24 (12.1)	104 (11.7)	
cT3	158 (79.8)	640 (71.8)	
cT4	14 (7.1)	144 (16.2)	
Clinical N stage, n(%)			0.030
cN negative	63 (31.8)	217 (24.4)	
cN positive	135 (68.2)	674 (75.6)	
Neoadjuvant chemotherapy regimen			0.683
Xeloda	95 (48.0)	389 (43.7)	
5-FU	66 (33.3)	321 (36.0)	
FL	32 (16.2)	162 (18.2)	
Others	5 (2.5)	19 (2.1)	
Neoadjuvant radiotherapy dose			0.774
5040Gy	172 (86.9)	790 (88.7)	
5400Gy	6 (3.0)	24 (2.7)	
Others nCRT-operative inveraval (days)	20 (10.1) 54±7	77 (8.6) 54±11	0.710
Postoperative chemotherapy, n(%)	175 (88.4)	836 (93.8)	0.007
Pretreatment Cell differentiation, n(%)			<0.001
Well differentiated	90 (45.4)	132 (14.8)	
Moderately differentiated	95 (48.0)	671 (75.3)	
Poorly differentiated	10 (5.1)	26 (2.9)	
Signet ring cell carcinoma	2 (1.0)	7 (0.8)	
Mucinous carcinoma	1 (0.5)	55 (6.2)	
Pathologic T stage, n(%)			<0.001
ypT0	198 (100.0)	15 (1.7)	
ypT1	0 (0.0)	49 (5.5)	
ypT2	0 (0.0)	312 (35.0)	
ypT3	0 (0.0)	492 (55.2)	
ypT4	0 (0.0)	23 (2.6)	
Pathologic N stage, n(%)			<0.001
ypN0	198 (100.0)	574 (64.4)	
ypN1	0 (0.0)	237 (26.6)	
ypN2	0 (0.0)	80 (9.0)	
Tumor regression grade, n(%)			<0.001
No	0 (0.0)	8 (0.9)	
Minimal	0 (0.0)	276 (31.0)	
Moderate	0 (0.0)	600 (67.3)	
Near complete	0 (0.0)	7 (0.8)	
Complete	198 (100.0)	0 (0.0)	
Lymph node harvest, n(%)			0.006
>12	135 (68.2)	513 (57.6)	
≤12	63 (31.8)	378 (42.4)	
Mean tumor size (cm)	2.0±1.0	2.7±1.5	<0.001
CRM involvement, n(%)	0 (0.0)	43 (4.8)	<0.001
Distal resection involvement, n(%)	0 (0.0)	2 (0.2)	0.669
TME grade, n(%)			0.453
Complete	197 (99.5)	889 (99.8)	
Incomplete	1 (0.5)	2 (0.2)	
Lymphatic invasion, n(%)	0 (0.0)	128 (14.4)	<0.001
Perineural invasion, n(%)	0 (0.0)	107 (12.0)	<0.001
Vascular invasion, n(%)	0 (0.0)	83 (9.3)	<0.001

*BMI* body mass index, *CEA* carcinoembryonic antigen, *AV* anal verge, *5-FU* fluorouracil, *FL* fluorouracil/leucovorin, *nCRT* neoadjuvant chemoradiotherapy, *CRM* circumferential resection margin, *TME* total mesorectal excision.

In terms of perioperative outcomes, as shown in Table 2, there were no significant differences between the two groups.

**Table 2:** Perioperative results of the patients according to the pCR.

	ypCR (n = 198)	Non-ypCR (n = 891)	*P*
Surgical approach			0.830
Open	134 (67.7)	610 (68.5)	
MIS	64 (32.3)	281 (31.5)	
Name of operation			0.055
LAR	175 (88.4)	721 (80.9)	
ISR	7 (3.5)	65 (7.3)	
APR	14 (7.1)	78 (8.8)	
Hartmann operation	2 (1.0)	27 (3.0)	
Mean operation time (min)	178±60	183±70	0.359
Diverting stoma, n(%)	91 (46.0)	410 (46.0)	1.000
Open conversion, n(%)	2 (1.6)	10 (1.6)	1.000
Intraoperative transfusion, n(%)			0.019
(+)	1 (0.5)	33 (3.7)	
(-)	197 (99.5)	858 (96.3)	
Length of stay (days)	11±8	12±8	0.699
Postoperative complications, n(%)	55 (27.8)	259 (29.1)	0.848
Surgical complications	51 (92.7)	237 (91.5)	
Non-surgical complications	2 (3.6)	8 (3.1)	
Both	2 (3.6)	14 (5.4)	
Surgical complications			
Anastomotic leakage	11 (5.6)	58 (6.5)	0.618
Rectovaginal fistula	3 (1.5)	10 (1.1)	0.715
Postoperative ileus	15 (7.6)	83 (9.3)	0.439
Urinary retention	10 (5.1)	61 (6.8)	0.427
Superficial surgical site infection	13 (6.6)	51 (5.7)	0.649
Intraabdominal bleeding	3 (1.5)	12 (1.3)	0.743
Intraluminal bleeding	1 (0.5)	10 (1.1)	0.700
Clavien-Dindo classification			1.000
I-II	41 (74.5)	193 (74.5)	
III-IV	14 (25.5)	66 (25.5)	
Postoperative mortality (<30days), n(%)	0 (0.0)	3 (0.3)	0.547

*CR* complete response, *MIS* minimally invasive surgery, *LAR* low anterior resection, *ISR* intersphincteric resection, *APR* abdominoperineal resection.

As can be seen from Table 2, there was also no significant difference in surgical procedures, such as open or minimally invasive surgery, operation time, and rate of diverting stoma between the two groups.

### Survival according to response to neoadjuvant treatment and pCR

**Figure 1:**
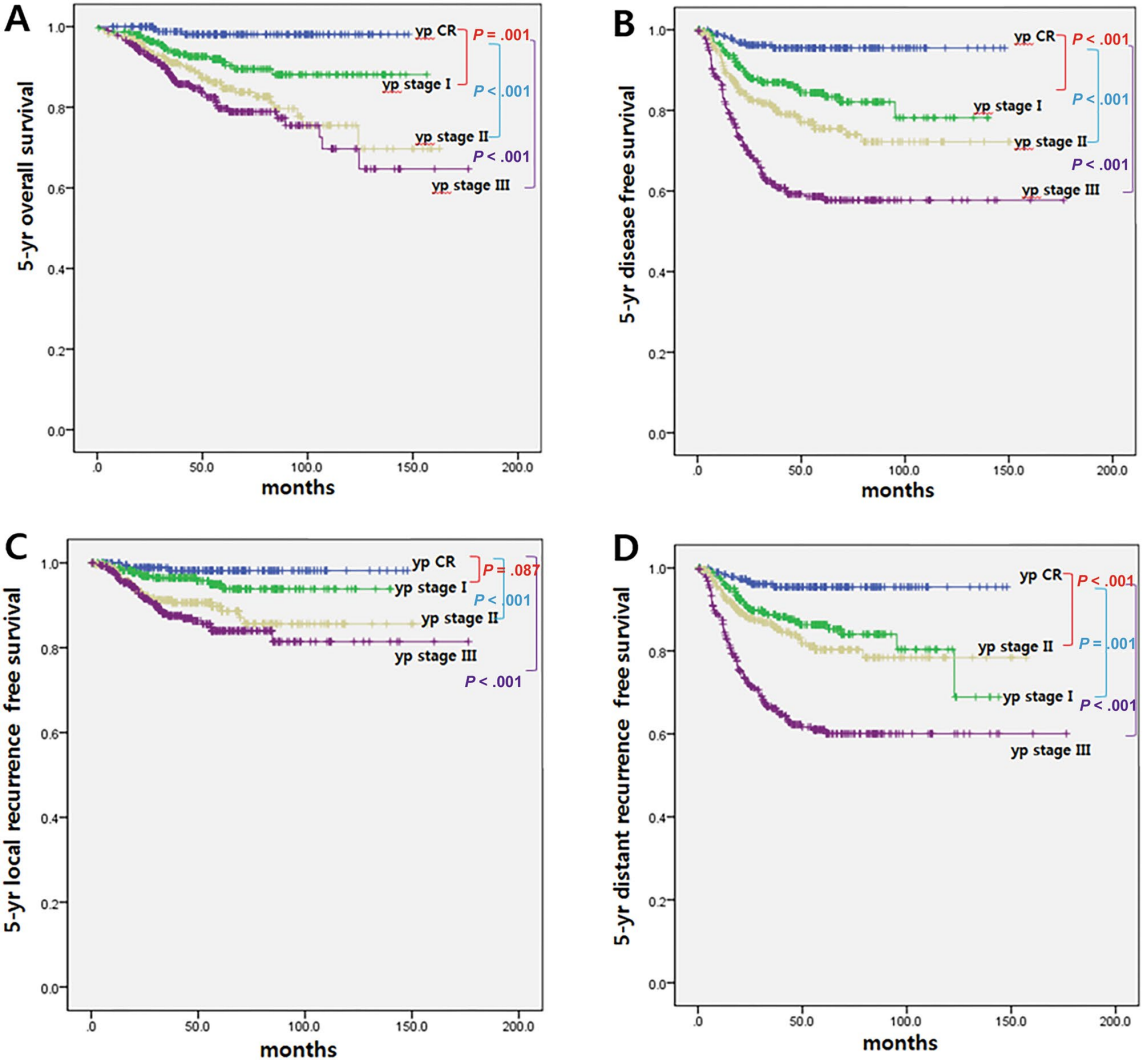
Survival according to response to neoadjuvant treatment. (**A**) 5-yr overall survival (**B**) 5-yr disease free survival (**C**) 5-yr local recurrence free survival (**D**) 5-yr distant recurrence free survival.

Figure 1 shows the survival rates according to the ypStage. The 5-yr overall survival (OS), 5-yr disease-free survival (DFS), and 5-yr local recurrence-free survival (LRFS) showed statistically significant differences by ypStage. To identify the impact of pCR on oncologic outcomes, we analyzed 5-yr OS, 5-yr DFS, 5-yr LRFS, and 5-yr distant recurrence-free survival (DRFS) rates according to ypStage. In the pCR group, 5-yr OS was 98.1%, 5-yr DFS was 95.5%, 5-yr LRFS was 98.2%, and 5-yr DRFS was 95.4%, which were the highest statistically significant values compared to those of other ypStages.

### Prognostic factors of OS and DFS

Univariate and multivariate analyses were performed to evaluate the value of pCR as an independent prognostic factor with respect to 5-yr OS and 5-yr DFS. From univariate analysis (Table 3), factors associated with poor overall survival included age ≥ 65 years, high preoperative CEA level, clinical node positivity, non-pCR, ypII–III, poor histology, and perineural invasion. In multivariate analysis, high preoperative CEA level, clinical node positivity, non-pCR, ypII–III, poor histology, and perineural invasion were associated with poor overall survival.

**Table 3:** Prognostic factors of OS, DFS, and LRFS.

Factors	Overall survival			Disease free survival			Local recurrence free survival		
	Univariate	Multivariate		Univariate	Multivariate		Univariate	Multivariate	
	*P*	HR (95% CI)	*P*	*P*	HR (95% CI)	*P*	*P*	HR (95% CI)	*P*
Preoperative CEA (mg/ml)									
≥5 versus <5	0.036	2.727 (1.630-4.563)	<0.001	0.022	3.269 (1.078-9.920)	0.036	0.022	1.071 (0.582-1.969)	0.825
Age (years)									
≥60 versus <60	0.035	1.585 (1.112-2.258)	0.011	0.272			0.272		0.334
Surgical approach								1.150 (0.866-1.527)	
Open versus MIS	0.150			0.882			0.882		
Type of operation									
SSS versus Non-SSS	0.563			0.294			0.294		
Gender									
Female versus male	0.212			0.642			0.642		
Clinical T stages									
2 versus 1	0.937			0.954			0.954		
3 versus 1	0.946			0.949			0.949		
4 versus 1	0.928			0.946			0.946		
Clinical N stages									
Positive versus negative	0.141			0.703			0.703		
Pathologic CR									
No versus yes	0.046	9.973 (3.171-31.366)	<0.001	0.037	6.972 (3.442-14.120)	<0.001	0.037		<0.001
ypII-III versus yp0-I	0.817			0.282			0.282		
Cell differentiation								6.049 (2.981-	
PD/MUC/SRC versus	0.003	3.311 (2.180-5.027)	<0.001	0.003	1.665 (1.127-2.459)	0.010	0.455	12.273)	
WD/MD									
Lymphatic invasion									
Yes versus no	0.616			0.062			0.062		
Venous invasion									
Yes versus no	0.732			0.350			0.350		
Perineural invasion									
Yes versus no	0.022	2.583 (1.576-4.234)	<0.001	0.035	2.848 (2.032-3.991)	<0.001	0.035		<0.001
CRM involvement									
Yes versus no	0.524			0.050	2.307 (1.387-3.838)	0.001	0.058	2.858 (1.995-4.095)	0.498
Lymph node harvest	0.297			0.025	0.900 (0.691-1.173)	0.437	0.025		
>12 versus ≤12								0.907 (0.684-1.203)	
Postoperative complications	0.899			0.805			0.805		
CDC III-IV versus I-II									
Adjuvant treatment									
Yes versus no	0.160			0.346			0.346		

*LRFS* local recurrence free survival, *CEA* carcinoembryonic antigen, *CR* complete response, *PD* poorly differentiated, *MUC* mucinous carcinoma, *SRC* signet ring cell carcinoma, *WD* well differentiated, *MD* moderately differentiated, *CRM* circumferential resection margin, *MIS* minimally invasive surgery, *SSS* sphincter saving surgery, *CDC* Clavien-Dindo Classification.

The results were similar with respect to DFS. In multivariate analysis, high preoperative CEA level, non-pCR, poor histology, lymphatic invasion, perineural invasion, and CRM involvement were associated with poor DFS (Table 3).

### Predictive factors of pCR and ypIII

Several pretreatment clinical factors were analyzed to identify the predictive factors of pCR (Table 4). Factors that were significantly associated with the achievement of a pCR were smaller tumor size (< 4 cm), clinical node negativity, and well-differentiated adenocarcinoma.

**Table 4:** Predictor factors of ypCR and yp stage III.

	ypCR			yp stageIII		
	Univariate	Multivariate		Univariate	Multivariate	
	*p*	OR (95% CI)	*p*	*p*	OR (95% CI)	*p*
Initial CEA (ng/l)						
≥5 versus <5	0.306			0.046	1.809 (1.041-3.144)	0.035
Age (years)						
≥60 versus <60	0.238			0.894		
Gender						
Female versus male	0.594			0.260		
Mean height from AV (cm)						
≥5 versus <5	0.491			0.306		
Pretreatment tumor size (cm)						
	<0.001	0.228 (0.136-0.383)	<0.001	<0.001	2.010 (1.508-2.679)	<0.001
≥4 versus <4						
Clinical T stages	0.189			0.107		
2 versus 1	0.559			0.306		
3 versus 1 4 versus 1	0.080			0.751		
Clinical N stages Positive versus negative	0.010	0.690 (0.493-0.965)	0.030	<0.001	2.200 (1.570-3.081)	<0.001
Cell differentiation	<0.01	0.027 (0.004-0.196)	<0.001	<0.001	3.461 (2.236-5.355)	<0.001
MD versus WD	0.004	0.128 (0.018-0.939)	0.043	<0.001	7.538 (3.488-16.295)	
PD versus WD			0.005			<0.001
SRC versus WD	0.001	0.047 (0.006-0.389)	0.033	0.010	6.031 (1.522-23.899)	0.011
MUC versus WD	0.001	0.064 (0.005-0.796)		<0.001	7.538 (3.878-14.653)	<0.011

*CR* complete response, *CEA* carcinoembryonic antigen, *AV* anal verge, *WD* well differentiated, *MD* moderately differentiated, *PD* poorly differentiated, *MUC* mucinous carcinoma, *SRC* signet ring cell carcinoma.

**Figure 2:**
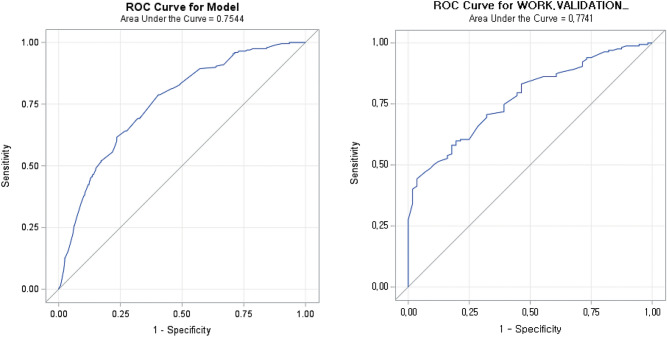
ROC curve for ypCR and validation model.

**Figure 3:**
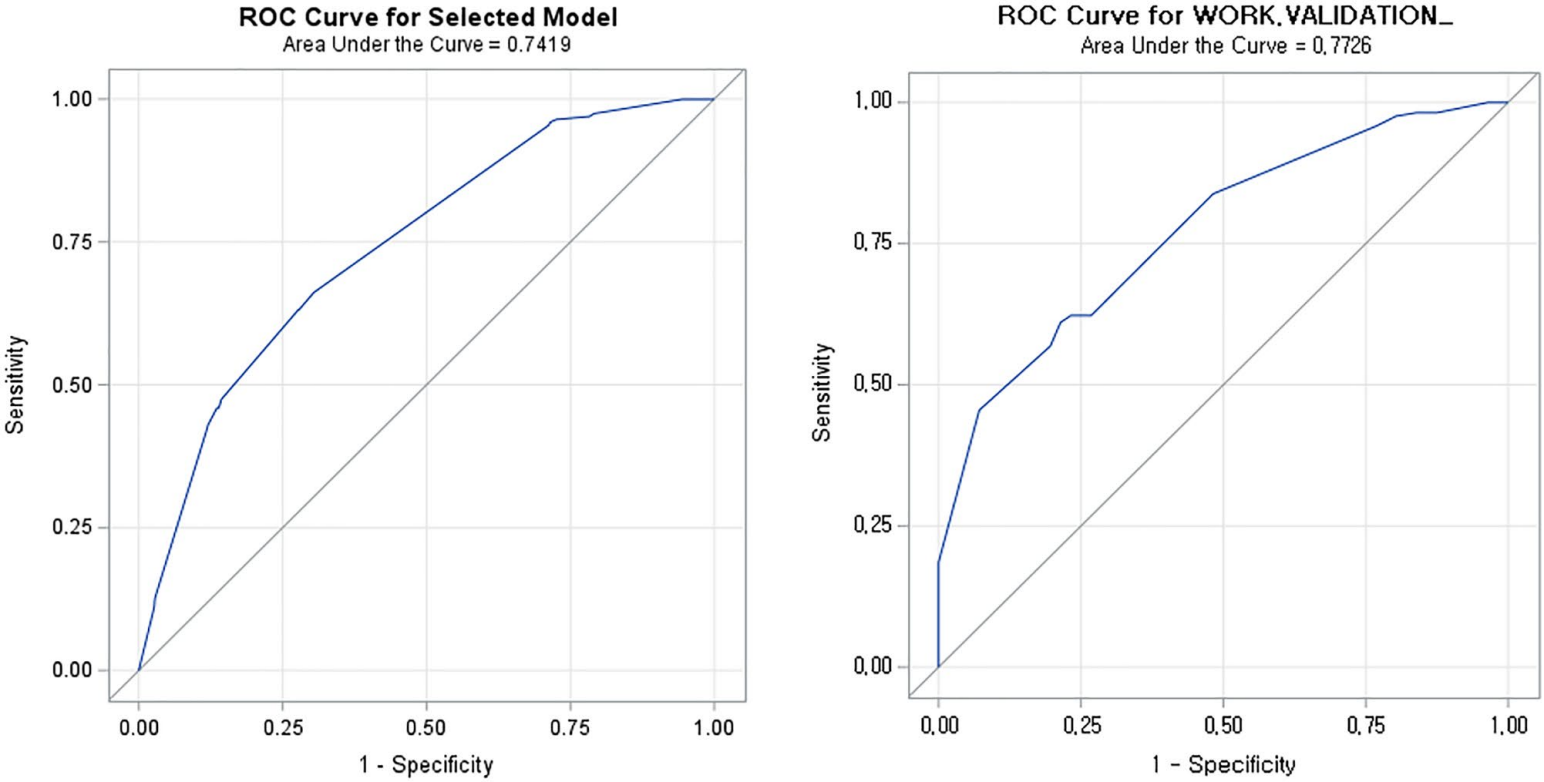
ROC curve for ypCR and validation model. Well differentiated and moderately differentiaed into groups.

Univariate and multivariate analyses were also performed to evaluate the risk factors associated with ypIII. On univariate analysis (Table 4), factors associated with ypIII included high preoperative CEA level, tumor size > 4 cm, clinical node positivity, and poor histology. Figure 2 shows the ROC curve based on factors such as age (60 years), sex, preoperative CEA level, clinical T and N stage, tumor location (AV 5 cm), tumor size (4 cm), and cell differentiation as well as the graph for application to the validation model (Fig. 2). We also grouped well differentiated and moderately differentiated together to analyze the prediction model and apply it to the validation model. The AUC value decreased slightly, but similar results were shown (Fig. 3).

### Patterns of recurrence in pCR

Eight (4.0%) out of 198 patients with pCR had recurrence. Local recurrence, on the lateral pelvic side wall, occurred in 1 patient; distant metastasis occurred in 6 patients (5 lung metastases, 1 liver metastasis, and 1 bone metastasis); and concurrent local recurrence and distant metastasis in 1 patient. The characteristics of these 8 patients are summarized in Table 5.

**Table 5:** Details of the 8 patients with recurrence after achieving pCR.

Age	Sex	Clinical staging	Tumor location from AV (cm)	CTx	RTx dose (Gy)	nCRT-surgery interval (wks)	Surgery	No.of harvested LNs	Cell diff	Adjuvant CTx	Location of recurrence	Treatment after recurrence	RFS (mon)	Death	OS (mon)	Current status
72	M	T4N1	1	5-FU	45.0	6	APR	4	WD	No	Pelvic/lung	CTx	17.0	Y	40.5	Death
76	M	T3N2	3	FL	50.4	6	ISR	10	WD	FL	Pelvic	No	14.4	N	69.2	Alive
51	M	T3N1	3	5-FU	50.4	6	ISR	6	MD	5-FU	Lung	Op, CTx	36.0	N	53.0	Alive
59	M	T3N1	6	Xeloda	50.4	7	LAR	13	MD	5-FU	Bone	RTx	5.4	Y	28.1	Death
65	M	T3N1	2	FL	50.4	6	ISR	8	WD	No	Lung	Op, CTx	11.7	N	36.8	Alive
58	M	T3N2	2	Xeloda	50.4	8	ISR	13	WD	No	Lung	No	6.3	N	7.7	Alive
72	M	T3N2	3	Xeloda	50.4	6	ISR	3	MD	No	Lung	Op	19.0	N	55.9	Alive
59	M	T3N2	5	FL	54.0	7	LAR	3	MD	FL	Liver	RFA	23.1	N	26.5	Alive

*CR* complete response, *AV* anal verge, *CTx* chemotherapy, *Op* operation, *5-FU* fluorouracil, *FL* fluorouracil/leucovorin, *RTx* radiotherapy, *nCRT* neoadjuvant chemoradiotherapy, *APR* abdominoperineal resection, *ISR* intersphincteric resection, *LAR* low anterior resection, d*iff* differentiation, *WD* well differentiated, *MD* moderately differentiated, *RFA* radiofrequency ablation, *RFS* recurrence free survival, *OS* overall survival.

## Discussion

In other studies, the rates of pCR after nCRT for rectal cancer are various from 10 to 30%^4,13,14^. In this study, the rate of non-metastatic rectal cancer patients who achieved pCR between 2000 and 2013 was 18.2%. We have identified several pretreatment clinical factors, such as tumor size ˂ 4 cm, clinical node negativity, and well-differentiated adenocarcinoma that can predict pCR. Several studies have reported various useful predictive factors for pCR, such as cell differentiation, tumor size, preoperative CEA level, and clinical T and N stages^14–18^. Understanding these factors can lead to the establishment of a treatment strategy for rectal cancer. Patients with high pCR achievement can consider local excision or the “watch and wait” strategy instead of a radical surgery. In contrast, more aggressive neoadjuvant treatment may be considered for patients with lower pCR prediction^3,10,11,19,20^.

In this study, univariate analysis revealed that clinical N-positive stage, tumor size ˃ 4 cm, and poorly differentiated tumors were significantly associated with lower odds of pCR and higher odds of ypIII. These results potentially assist in predicting response to nCRT and, on this premise, allow a more accurate prediction of patients likely to achieve pCR. Currently, “watch and wait” is not a routine procedure in our hospital. We have reserved “watch and wait” for patients with comorbidities or those with cCR, after sufficient explanation and informed consent, including the possibility of recurrence and frequent follow-up.

The present study also demonstrated better oncologic outcomes in patients who achieved pCR than in those who did not. This result corroborates those of previous studies, which reported that patients who achieved pCR showed better oncologic outcomes^3,4^. The finding that the prognosis of a patient with pCR is better than that of one without pCR suggests that it is important to achieve pCR. In patients who have predictive factors for poor response to nCRT, such as large tumor size, advanced clinical node stage, and aggressive histology, aggressive neoadjuvant treatment may be a preferred option, such as increased radiation dose or boosts or additional chemotherapy.

Although several patients achieved pCR after nCRT and radical surgery, some of them were at risk of recurrence, and, overall, recurrence occurred in 8 patients (4.0%) with pCR after TME in this analysis. One patient developed local recurrence, while distant metastases occurred in 6 patients, and one patient had both local and distant recurrence. The individual data of these 8 recurrent patients did not help in explaining these local and distant recurrences. It was difficult to analyze statistically significant prognostic factors because there were fewer cases of recurrence. However, we observed that distant metastases were the major recurrence pattern in patients who had achieved pCR after nCRT, which concurs with the findings of other studies^6,21,22^. The patients with recurrence had the following features: clinical T3–4 tumors and node positivity, tumor location from the anal verge lower than 5 cm, and history of intersphincteric resection. Four of the patients with recurrent metastases received adjuvant chemotherapy, and the other four did not. The role of adjuvant chemotherapy in patients with pCR following nCRT and radical resection is still not entirely clear. However, we consider administration of adjuvant chemotherapy for patients with these factors, even for those who have achieved pCR following nCRT and radical resection.

The limitations of this study include its retrospective and single-center design. Despite these limitations, to the best of our knowledge, this is the first study to propose predictive factors for pCR and predictive models based on ypCR. Furthermore, this study analyzed the oncologic outcomes of pCR, which could offer a treatment strategy for rectal cancer. Less invasive methods, such as “watch and wait,” may be considered for those who are predicted to achieve pCR, whereas more aggressive neoadjuvant treatment, including increased radiation dose or induction/consolidation chemotherapy, could be considered for patients at high risk of ypIII.

In conclusion, the factors significantly associated with the achievement of a pCR were smaller tumor size (< 4 cm), clinical node-negativity, and well-differentiated adenocarcinoma. Patients with pCR displayed better long-term outcomes than those with non-pCR. The pCR-prediction model, based on predictive factors, is potentially useful for prognosis and for prescribing a treatment strategy in patients with advanced rectal cancer who need nCRT.

## Patients and methods

Between January 2000 and December 2013, a total of 1,089 patients with primary rectal cancer received nCRT followed by radical resection at a single institution. Patients who had undergone radical resection were included if they had biopsy-proven adenocarcinoma of the rectum ≤ 10 cm from the anal verge. Patients were excluded if they had recurrent or metastatic cancer, previous chemotherapy or pelvic radiotherapy, hereditary rectal cancer, or local excision. All methods were performed in accordance with the relevant guidelines and regulations. This study was approved by the Institutional Review Board (IRB) of Samsung Medical Center, Sungkyunkwan University School of Medicine (IRB No. SMC 2019-10-134-001). Since it was a retrospective study through medical charts, the need for written informed consent was waived by the IRB of Samsung Medical Center, Sungkyunkwan University School of Medicine.

All patients underwent preoperative staging with rectal magnetic resonance imaging and computed tomography scans of the abdominopelvic and thoracic cavities. Tumor size and lymph node metastasis were measured through rectum or pelvic MRI performed at the time of diagnosis. The size of tumor was measured by longitudinal tumor size on MRI. Lymph node metastasis was determined based on size, irregular margin, and heterogenic signal intensity. The above results were officially reported by a radiologist specializing in colorectal cancer. Patients with clinical T3 and higher or with clinical nodal involvement received nCRT. The chemoradiation regimen consisted of long-course radiation with 4500–5400 cGy over 5–6 weeks with synchronous intravenous 5-fluorouracil, Xeloda, or FL (*FL* fluorouracil/leucovorin) chemotherapy. Surgery was performed–6–8 weeks after completion of chemoradiation. Postoperative chemotherapy after radical resection was recommended for all patients.

The macroscopic quality of TME specimens was assessed by a single pathologist specializing in colorectal disease immediate after surgery according to the grading system used by the American College of Surgeons Oncology Group Z6051^23^.

There are three pathologists specializing in colorectal cancer who analyze specimens with microscope and report the final pathological staging. A positive circumferential resection margin (CRM) was defined as a distance ≤ 1 mm between the deepest tumor invasion and the mesorectal fascia^24^.

The stage was determined according to the eighth edition of the American Joint Committee on Cancer staging manual^25^. A pCR was defined as the absence of viable cancer cells observed in the specimen after radical resection. We used the Dworak system to determine the degree of tumor regression, which ranges from GR 0 (abscess of regression) to GR 4 (complete regression)^26^.

### Statistical analysis

We used SPSS for Windows version 25.0 (SPSS, Chicago, IL, USA) for analysis. The chi-square test, Mann–Whitney U test, or Fisher’s exact test were used to analyze the differences between the two groups. The Kaplan–Meier method was used for survival rate analysis. Multivariate analyses were performed using the Cox proportional hazard model. Clinical factors were then subjected to stepwise multivariate logistic regression analysis. A *P* value less than 0.05 was considered to indicate statistical significance.
